# Idiopathic subglottic stenosis arises at the epithelial interface of host and pathogen

**DOI:** 10.21203/rs.3.rs-2945067/v1

**Published:** 2023-05-19

**Authors:** Alexander Gelbard, Meghan H. Shilts, Britton Strickland, Kevin Motz, Hsiu-Wen Tsai, Helen Boone, Wonder P. Drake, Celestine Wanjalla, Paula Marincola Smith, Hunter Brown, Marisol Ramierez, James B. Atkinson, Jason Powell, John Simpson, Seesandra V. Rajagopala, Simon Mallal, Quanhu Sheng, Alexander T. Hillel, Suman R. Das

**Affiliations:** Vanderbilt University Medical Center; Vanderbilt University Medical Center; Vanderbilt University Medical Center; Johns Hopkins; Johns Hopkins; Vanderbilt University Medical Center; Vanderbilt University Medical Center; Vanderbilt University Medical Center; Vanderbilt University Medical Center; Vanderbilt University Medical Center; The Newcastle upon Tyne Hospitals NHS Foundation Trust; Vanderbilt University Medical Center; Newcastle University; Newcastle University; Vanderbilt University Medical Center; Vanderbilt University Medical Center; Vanderbilt University Medical Center; Johns Hopkins; Vanderbilt University Medical Center

## Abstract

**Background:**

Idiopathic subglottic stenosis (iSGS) is a rare fibrotic disease of the proximal airway affecting adult Caucasian women nearly exclusively. Life-threatening ventilatory obstruction occurs secondary to pernicious subglottic mucosal scar. Disease rarity and wide geographic patient distribution has previously limited substantive mechanistic investigation into iSGS pathogenesis.

**Result:**

By harnessing pathogenic mucosa from an international iSGS patient cohort and single-cell RNA sequencing, we unbiasedly characterize the cell subsets in the proximal airway scar and detail their molecular phenotypes. Results show that the airway epithelium in iSGS patients is depleted of basal progenitor cells, and the residual epithelial cells acquire a mesenchymal phenotype. Observed displacement of bacteria beneath the lamina propria provides functional support for the molecular evidence of epithelial dysfunction. Matched tissue microbiomes support displacement of the native microbiome into the lamina propria of iSGS patients rather than disrupted bacterial community structure. However, animal models confirm that bacteria are necessary for pathologic proximal airway fibrosis and suggest an equally essential role for host adaptive immunity. Human samples from iSGS airway scar demonstrate adaptive immune activation in response to the proximal airway microbiome of both matched iSGS patients and healthy controls. Clinical outcome data from iSGS patients suggests surgical extirpation of airway scar and reconstitution with unaffected tracheal mucosa halts the progressive fibrosis.

**Conclusion:**

Our data support an iSGS disease model where epithelial alterations facilitate microbiome displacement, dysregulated immune activation, and localized fibrosis. These results refine our understanding of iSGS and implicate shared pathogenic mechanisms with distal airway fibrotic diseases.

## INTRODUCTION

Idiopathic subglottic stenosis (iSGS) is a rare (Maldonado et al., 2014) but devastating fibroinflammatory airway disease that occurs almost exclusively in adult Caucasian women (Gelbard et al., 2015). The disease is characterized by mucosal inflammation and localized fibrosis resulting in life-threatening blockage of the upper airway (Gelbard et al., 2016). Current treatments are limited by either their invasive nature or high recurrence rates, and the majority of iSGS patients require frequent procedural interventions following their initial diagnosis (Gelbard et al., 2019). Given the significant emotional, physical, and financial costs associated with recurrent airway obstruction (Gnagi et al., 2015), most research efforts have focused on procedural techniques to improve airway patency (Daniero et al., 2017). However, highly focused scientific approaches to identify key elements of iSGS disease pathophysiology are essential to developing less invasive and more durable treatments.

Histologically, iSGS cases show pronounced fibrosis restricted to the proximal airway mucosa (Mark et al., 2008). Diverse diseases in divergent organ systems are associated with fibrosis, suggesting common biologic triggers (Wynn and Ramalingam, 2012). In alternate pulmonary pathologies, airway fibrosis has been linked to structural and functional disruption of the respiratory epithelium. Alterations in apical cell-cell junctions increase epithelial permeability and enhance translocation of pathogens into the subepithelial space, where they encounter local immune cells and generate airway inflammation culminating in tissue fibrosis (Rezaee and Georas, 2014). Recent data suggests iSGS may share these pathogenic mechanisms with lower airway fibrotic diseases. Altered epithelial morphology (Lina et al., 2022) and chronic inflammation in airway scar (Clark, 2015) provide preliminary support to the hypothesis that in iSGS, epithelial dysfunction promotes an aberrant immune response culminating in tissue fibrosis.

In iSGS the functional alterations underlying airway remodeling remain poorly understood. In this study, we harnessed tissue samples from an international iSGS patient cohort and cutting-edge molecular tools to define the molecular phenotype of the proximal airway mucosa at single-cell resolution. Our results provide an unbiased assessment of the cell types within the normal human subglottis and illuminate the changes accompanying iSGS. The results suggest epithelial barrier dysfunction and immune infiltration are key components of iSGS pathogenesis. Matched superficial and deep tissue microbiomes support displacement of the native microbiome into the lamina propria of iSGS patients rather than disruption of bacterial community structure. However, animal models confirm that bacteria are necessary for pathologic proximal airway fibrosis and suggest an equally essential role for host adaptive immunity in remodeling after mucosal injury. Clinical data support the role of epithelial dysfunction in treatment response. Our data suggests that in iSGS the native microbiome is displaced across a dysfunctional epithelial barrier leading to an adaptive immune response which drives obstructive airway fibrosis. These novel results reshape our understanding of iSGS, implicate shared pathogenic mechanisms with distal airway fibrotic diseases, and open new avenues for therapy.

## RESULTS

**Figure 1:**
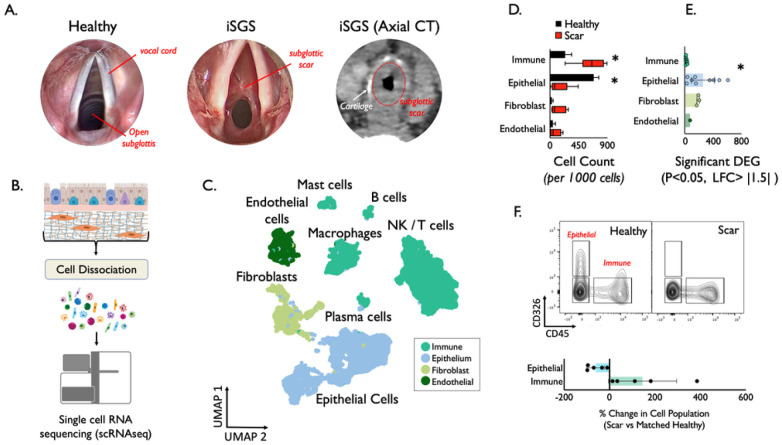
Anatomy of mucosal scar obstructing the subglottis in iSGS. Submucosal thickening with preserved cartilage seen on axial computed tomography (A). Workflow to generate single cell suspensions from subglottic scar (B). Uniform Manifold Approximation and Projection (UMAP) of jointly analyzed single-cell transcriptomes from 25,974 cells from 7 iSGS mucosal scar and 3 healthy mucosa annotated by cell type. (C) Cell types/states manually grouped into 4 broad tissue classes. (D) Quantification of cell types showed significantly increased immune cell populations in airway scar (p = 0.018) and significantly reduced epithelial cell numbers (p < 0.001) Boxes depict median and interquartile range wiskers show min to max, *P < 0.05 by Mann-Whitney U. (E) Number of differentially expressed genes (DEG) in each cell type in iSGS airway scar and paired healthy mucosa [negative binomial test, log fold change (FC) cutoff of 1.5 and adjusted P value of <0.05]. Representative flow cytometry of fresh single cell suspension from matched iSGS airway scar and healthy mucosa (n=5) confirming depletion of epithelial cells and increase in immune cells within iSGS airway scar when compared to matched healthy control. Graph depicting % change in cell population for matched scar/healthy mucosa for each of the five individual patients. Bar represents mean, error bars SEM, and dots show individual patients (F).

Single-cell sequencing reveals epithelial cell loss and a pronounced immune cell infiltrate in subglottic mucosal scar of iSGS patients. iSGS patients possess obstructive mucosal scars in the proximal airway below the vocal cords (Fig. 1A). Study patients were diagnosed according to standard clinical criteria (Gelbard et al., 2015). Tissue biopsies obtained under sterile conditions in the OR, were immediately digested into single-cell suspensions for single-cell RNA sequencing (Fig. 1B). To determine the distribution and phenotype of the cellular populations present in iSGS airway scar, we generated single-cell suspensions from tissue biopsies of both airway scar (n = 7). We matched unaffected airway mucosa (n = 3) (**Supplemental Table T1**) and performed scRNAseq using the 10x Genomics Chromium platform (see supplementary Materials and Methods). The samples were collected and processed at two different sites (**Supplemental Table T2**); however, both sites collected cases and controls. To maximize our ability to identify rare cell populations, we jointly analyzed data from all samples. We defined inclusion criteria for cells based on observations from the entire dataset, removed low-quality cells accordingly, applied normalization and variance stabilization of the 25,974 recovered cells using Seurat(Hafemeister and Satija, 2019; Stuart et al., 2019), integrated the data using the harmony(Korsunsky et al., 2019), performed unsupervised clustering using Seurat, and classified the cell type of each cluster based on PanglaoDB(Franzen et al., 2019) followed by manual annotation based on canonical markers to annotate clusters. We defined 22 cell types/states in the subglottis **(Supplemental Figure S1**). Initially one small CD8 effector T cell population was grouped together with a larger CD8 T effector population due to observations that cell cycle activity was driving distinct cluster identity. All cell types were identified both in airway scar and healthy mucosa. Notably, we did not observe overt batch effects driven by processing site or sequencing batch in our dimensionality reduction and visualization (**Supplemental Figure S2 & S3**). Cell types/states were also manually grouped into 4 broad tissue classes (Immune/Epithelial/Endothelial/Mesenchymal) based on their identity (Fig. 1C) and confirmed with canonical lineage markers (**Supplemental Figure S4**). Quantification of cell types demonstrated significant differences between iSGS airway scar and matched healthy mucosa controls. Airway scar showed significantly more Immune cells (cell count per 1000 cells: scar vs healthy control: 636 vs. 238, P = 0.018) and significantly fewer epithelial cells (scar vs healthy control: 155 vs. 685, P < 0.001) (Fig. 1D). In addition to quantitative differences in cell types/states, we compared phenotypic alterations between scar and healthy mucosa by examining the number of differentially expressed genes using EdgeR(Robinson et al., 2010) (DEG: P < 0.05, log fold change > |1.5|). This analysis demonstrated wide variability across the cell types, with epithelial cells and fibroblasts showing the greatest number of DEG (**Supplemental Figure S5**). When grouping cells into their tissue layer, the epithelium demonstrated significantly more DEG than immune cells (mean number of genes P < 0.05 and fold change > |1.5|, Epithelium = 252 vs 11 in immune, P = 0.007) (Fig. 1E). The difference between epithelial cells and fibroblasts or endothelium was not significant. These results suggest that in addition to a quantitative reduction in cell numbers, the residual epithelium in iSGS scar is also phenotypically distinct. The cell type quantification based on transcriptional data was confirmed at the protein level with flow cytometry (Fig. 1F).

**Figure 2:**
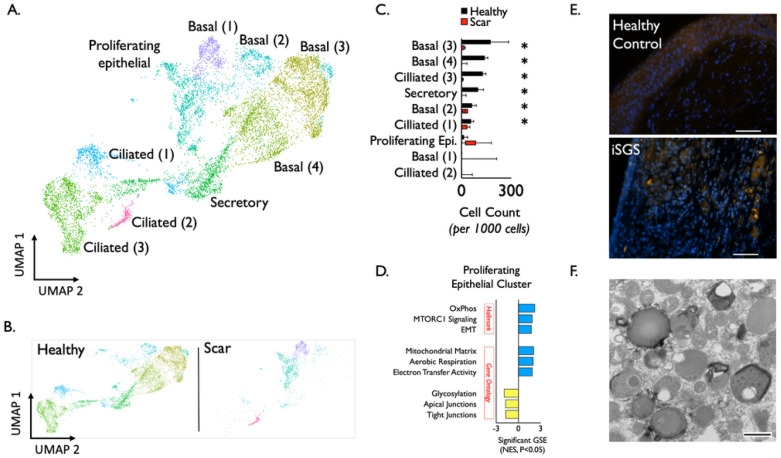
Detailed analysis of epithelial clusters (A) identified conserved transcriptional programs in basal (four clusters), ciliated (three clusters), secretory (one cluster), and a proliferating cell subset (one cluster) in both healthy and scar (B). Quantification of epithelial cell types/states show significant reduction in clusters comprising basal, secretory, and ciliated cells in scar samples (Boxes depict median and interquartile range, whiskers show min to max, *P < 0.05 by Mann-Whitney U) (C). In addition to the dramatic loss of basal cells within airway scar, geneset enrichment analysis for *Hallmark genesets* and *Gene Ontology Biological Processes* genesets demonstrate that residual proliferating epithelial cells express a molecular program for EMT. Blue represents genesets upregulated in scar epitheluim, yellow represents genesets down regulated in scar epithelium compared to healthy mucosa. FISH with the pan-bacterial probe Eub338 shows iSGS mucosa possessed signal for bacteria in the deeper lamina propria while healthy control did not. Scale bar represents 50μm (E). Transmission electron microscopy of separate iSGS scar specimen demonstrated numerous forms consistent with the size and shape of bacteria in the cell cytoplasm. Scale bar represents 500nm (F). mTORC1: mechanistic target of rapamycin complex 1, OxPhos: oxidative phosphorylation, EMT: epithelial-mesenchymal transition. FISH: Fluorescence in situ hybridization.

Molecular and functional evidence of epithelial dysfunction in iSGS scar. We further analyzed the epithelial clusters (Fig. 2A) and identified conserved transcriptional programs in basal (four clusters), ciliated (three clusters), secretory (one cluster), and a proliferating cell subset (one cluster). Based on our observation of differential cluster abundance between scar and healthy mucosa (Fig. 2B), we quantified the number of cell types/states from both scar and healthy mucosa. The clusters comprising basal, secretory, and ciliated cells showed significant reductions in scar samples (Boxes depict median and interquartile range, whiskers show min to max, *P < 0.05 by Mann-Whitney U.; Fig. 2C). In addition to the dramatic loss of basal cells within airway scar, geneset enrichment analysis demonstrated that residual proliferating epithelial cells expressed a molecular program for epithelial-mesenchymal transition (EMT) (Fig. 2D). Additional upregulated genesets included oxidative phosphorylation and mTOR signaling consistent with observed proliferation markers Regulatory Subunit 105 (Ki67) and Cyclin-Dependent Kinase 1 (CDK1) (**Supplemental Figure S4**). Gene ontology (GO) pathway analysis supported the Hallmark geneset EMT findings; iSGS airway scar showed enrichment for mitochondrial matrix genes (along with aerobic respiration and electron transfer activity). In parallel, proliferating epithelial cells in iSGS airway scar showed down-regulated glycosylation and junctional protein complexes (both apical and tight). Both aerobic respiration and loss of cell-cell adhesion are consistent with EMT.

To provide functional evidence of epithelial barrier dysfunction, we investigated if native bacterial displacement into the deeper lamina propria was a unique feature of iSGS. Employing fluorescence in situ hybridization (FISH) with the pan-bacterial probe, Eub338, we investigated if mucosal biopsies from iSGS and healthy controls evidenced bacteria in the deep layers of the proximal airway mucosa. Representative FISH stains show iSGS mucosa possessed signal for bacteria in the deeper lamina propria while healthy control did not (Fig. 2E). In a separate biopsy derived from iSGS mucosal scar, transmission electron microscopy demonstrated numerous forms consistent with the size and shape of bacteria in the cell cytoplasm, supporting the FISH staining (Fig. 2F).

**Figure 3:**
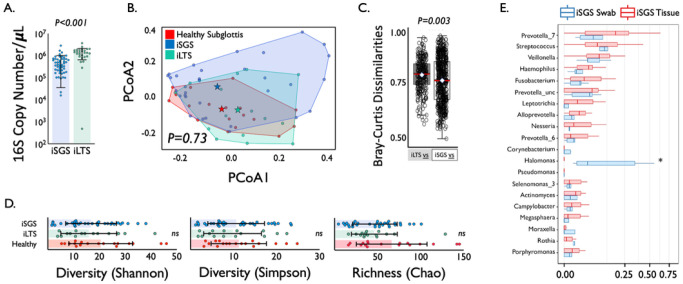
qPCR of 16S rRNA showed a significantly higher bacterial load (copy number per μL) in iLTS patients (green) compared to iSGS samples (blue) (P<0.0001) (A). Principal coordinate analysis of proximal microbiome in iSGS, post-intubation subglottic stenosis (iLTS), and healthy controls showing no significant differences in the centroids between the three groups (PerMANOVA adonis2 testing p = 0.06) (B). Binary comparisons between healthy and iLTS samples, and between heathy and iSGS samples using Bray-Curtis dissimilarities showed iSGS samples more closely resembled healthy controls (Wilcoxon rank sum test with continuity correction, p-value=0.001) (C). Microbial alpha diversity (Shannon and Simpson indicies), and richness (Chao1 index) shown for iLTS, iSGS, and healthy control samples (D). Individual sample values are represented as dots. Group mean depicted by box center line, and standard deviation represented by error bars. Microbial alpha diversity and richness were not significantly different between cases and controls. Comparison of bacterial abundance between sampling via mucosal swab vs. tissue biopsy in iSGS patients. The top 20 most abundant genera detected via the two methods with the genera abundance represented by boxplots; the median is represented by the center line of the box, and the interquartile range is represented by the upper and lower edges of the box. The vertical lines represent the whiskers (minimum and maximum, excluding outliers); outliers are represented as dots. Blue boxes are samples from Hillel et al., where the iSGS microbiome was sampled using swabs (N=5). Red boxes are samples from the tissue samples (N=37). Of the top 20 most abundant genera, the only significantly different genus between the two studies was *Halomonas*, which was relatively abundant in the iSGS swab samples but was not found in the iSGS tissue samples (E).

Characterization of the mucosal microbiome in iSGS. Given the observed bacterial displacement into the lamina propria of the airway mucosa, we investigated if alterations in microbiome community structure were also observed in iSGS. We first quantified the number of 16S rRNA gene copies in deep tissue biopsies via qPCR (Fig. 3A). We detected consistent signals in both iSGS patients and disease controls (patients that developed subglottic stenosis following prolonged intubation: iLTS). iLTS patients had a significantly higher bacterial load than iSGS samples (mean iSGS copy number: 520,000 vs. iLTS: 1,370,000; P < 0.0001). We then performed 16S rRNA sequencing for insight into bacterial community structure. For further analysis, 37/50 (74%) iSGS and 18/27 (67%) iLTS samples were retained after implementing a cutoff of 500 high-quality 16S reads (2 standard deviations above the maximum number of reads in any of the negative controls).

The top 20 most abundant bacterial families and genera are shown per each sample for iSGS, iLTS and healthy subglottic controls (**Supplemental Figure S6**). The majority of bacterial species present in healthy subglottis were consistent with the established healthy lung microbiome composed of supraglottic predominant taxa (e.g., *Prevotella*, *Streptococcus*)(Kitsios et al., 2018). Using principal coordinates analysis (PCoA), we compared the overall microbial community structure between iSGS, iLTS, and healthy subglottic controls. As seen in Fig. 3B, there was no significant differences in the centroids between the three groups (PerMANOVA adonis2 testing p = 0.06). To validate these findings, we next utilized Bray-Curtis dissimilarities to make binary comparisons between healthy and iLTS samples and between healthy and iSGS samples. iSGS samples more closely resembled healthy controls than iLTS samples resembled healthy subglottic controls (Wilcoxon rank sum test with continuity correction, p-value = 0.001 Fig. 3C).

Additional microbial community structure testing using established diversity and richness indices confirmed no detectable differences between iSGS, iLTS and healthy controls. ANOVA testing of alpha diversity (mean Shannon index - iSGS: 15.28 vs iLTS: 14.53 vs healthy controls; p = 0.39, mean Simpson index - iSGS: 15.28 vs iLTS: 14.53 vs healthy controls; p = 0.822) and richness (mean Chao1 index - iSGS: 40.75 vs iLTS: 40.45; vs. healthy controls, p = 0.082, Fig. 3D) showed no significant differences. There was not a significant association between iSGS disease severity and overall bacterial load (p = 0.36, **Supplemental Fig. 7A**) nor between disease severity and alpha diversity (p = 0.453, **Supplemental Fig. 7B**) or richness (p-value = 0.6078, data not shown).

Anatomic location of bacterial species in iSGS mucosal scar. To validate our findings and explore if superficial and deep tissue sampling methods produced unique bacterial communities, we next compared published 16S rRNA sequencing data of superficial swabs of iSGS scar (n = 5)(Hillel et al., 2019) with our deep tissue biopsies (Fig. 3E). The top 20 most abundant genera were highly concordant between the superficial and deep sampling methods, despite differences in patient populations and lab processing protocols; offering support for our findings (the one significantly different genus was *Halomonas* which was abundant is swab samples and absent in tissue).

**Figure 4:**
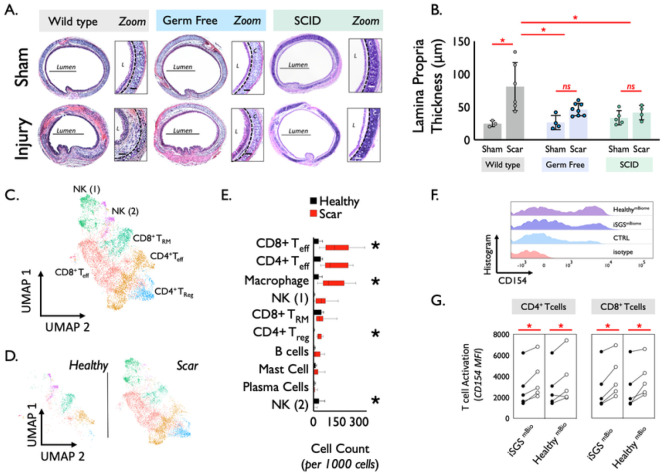
Established murine model of subglottic stenosis (A) demonstrated significant thickening of the lamina propria 14 days after epithelial injury in wild type mice (p=0.0036). However, no significant thickening of the lamina propria was observed in either germ free mice (p=0.56), or severe combined immunodeficient mice (SCID) (p=0.98) Wild type injury was significantly greater than both germ-free injury (p=0.025) and SCID injury (p=0.036) (B). Immune cell identification and functional characterization in iSGS Proximal Airway Scar via UMAP of jointly analyzed single-cell transcriptomes (C) Analysis of immune clusters identified conserved transcriptional programs in T cells (four clusters) and NK cells (two clusters), with differntial abundance in healthy mucosa and airway scar (D). Quantification of immune cell types in iSGS airway scar versus matched healthy mucosa. Boxes depict median and interquartile range wiskers show min to max, *P < 0.05 by Mann-Whitney U. (E). Single cell suspensions from 5 unique iSGS patients cultured in the presence of matched iSGS airway microbiome, the microbiome from an unrelated healthy subject, or left untreated. 24 hours after stimulation, expression levels of activation marker CD154 were quantified on CD4+ and CD8+ T cells. (F) Both the matched iSGS microbiome, as well as the microbiome from an unrelated healthy control significantly up-regulated CD154 when compared to untreated experimental controls (CD4+ matched iSGS microbiome vs untreated: p=0.007; CD4+ unrelated healthy microbiome vs untreated: p=0.04), (CD8+ matched iSGS microbiome vs untreated: p=0.005; CD8+ unrelated healthy microbiome vs untreated: p=0.03). No significant difference observed between cells treated with a matched iSGS microbiome or cells treated with an unrelated healthy microbiome (G).

Both bacteria and adaptive immunity are necessary for mucosal scarring after epithelial injury in an animal model of subglottic stenosis. We next employed an established murine model of subglottic stenosis (Hillel et al., 2014) to investigate the roles of bacteria and adaptive immunity in the mucosal fibrosis that characterizes iSGS (**Supplemental Fig. 8**). As expected, wild-type mice developed mucosal inflammation and significant thickening of the lamina propria 14 days after injury (WT injury vs WT sham: 81μm +/− 37 vs. 24μm +/− 4, p = 0.0036). Interestingly however, no significant thickening of the lamina propria was observed when using either germ-free mice (GF injury vs GF sham: 46μm +/− 9.4 vs. 27 +/− 11, p = 0.56), or severe combined immunodeficient mice (SCID)(Vladutiu, 1993) lacking an adaptive immune response (SCID injury vs SCID sham: 41μm +/− 11 vs. 34 +/− 11, p = 0.98). WT injury was significantly greater than both GF injury (p = 0.025) and SCID injury (p = 0.036) (Figs. 4A & B).

iSGS scar demonstrates increased adaptive immune cell subsets. Next, to better define the constituents of the immune cell infiltrate seen in iSGS airway scar (Fig. 4C), we compared the cell types observed in the scar with unaffected mucosa (Fig. 4D). Quantification of cell types demonstrated significantly greater CD8 T_eff_ cells in scar (scar vs. healthy: 167+/−103 vs. 34+/26, P = 0.033), along with more CD4 + T_reg_ (scar vs. healthy: 37+/−15 vs. 4+/−3 P = 0.005) and more NK cluster 2 cells (scar vs. healthy: 57+/−54 vs. 5+/−5, P = 0.025). In contrast, the NK cluster 1 population was significantly reduced in scar (scar vs. healthy: 4+/−9 vs. 36+/−37) (Fig. 4E).

The native proximal airway microbiome generates an antigen-specific immune response in infiltrating CD4 + and CD8 + T cells. In order to probe the function of the observed immune infiltrate, tissue biopsies acquired during the operative endoscopy of 5 unique iSGS patients were used to create fresh single-cell suspensions as described. Suspensions were rested for 6 hours, then cultured in the presence of a matched iSGS airway microbiome, the microbiome from an unrelated healthy subject, or left untreated.

After 24 hours of stimulation, cells were washed and stained for markers of T cell activation (CD154) and analyzed via flow cytometry (Fig. 4F). For CD4 + and CD8 + T cells, both the matched iSGS microbiome, as well as the microbiome from an unrelated healthy control, significantly upregulated CD154 when compared to untreated experimental controls (CD4 + matched iSGS microbiome vs. untreated: 3741 +/−1934, vs. 2912 +/− 1958, p = 0.007; CD4 + unrelated healthy microbiome vs. untreated: 3641+/− 2287 vs. 2912 +/− 1958, p = 0.04), (CD8 + matched iSGS microbiome vs. untreated: 3943 +/− 1989 vs. 2845 +/− 2098, p = 0.005; CD8 + unrelated healthy microbiome vs. untreated: 3784 +/− 1768 vs. 2845 +/− 2098, p = 0.03) (Fig. 4G). For both CD4 + and CD8 + T cells, CD154 expression was not significantly different between cells treated with a matched iSGS microbiome or cells treated with an unrelated healthy microbiome. While the native airway microbiome triggers an antigen-specific immune response in iSGS mucosa, T cell activation in scar can also occur when presented with the bacterial constituents of a healthy individual. Taken together with data showing the abundant bacteria in healthy proximal airway mucosa, these results reinforce the functional impact of the observed epithelial barrier dysfunction, allowing native pathogens to breach the mucosa barrier and trigger inappropriate host immune activation.

**Figure 5:**
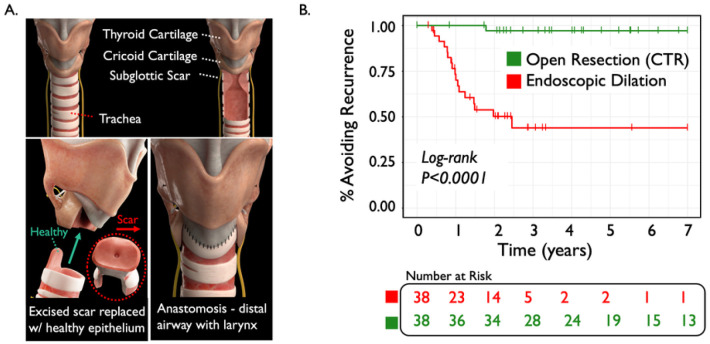
Technique of open resection (cricotracheal resection: CTR) for idiopathic subglottic stenosis. Anatomic landmarks of the Thyroid and Cricoid cartilage along with the defined rings of the trachea. Obstructing mucosal scar arises below the vocal cords at the junction of the cricoid cartilage and proximal trachea (A). Oblique view indicates removal of affected proximal trachea as well as anterior arch of cricoid but conservation of posterolateral laminae and posterior cricoid to preserve recurrent laryngeal nerves (nerves indicated in yellow). The mucosal stenosis is excised, and a broad flap of healthy tracheal mucosa is pulled into place from below to resurface the airway (B). The continuity of the airway mucosa is resotred at the completion of the procedure (C). Kaplan-Meyer survival analysis showing patients undergoing CTR had a significantly lower rate of disease recurrence (Log-rank, P<0.0001) (D).

Clinical data suggest that restoring epithelial barrier function is associated with a more durable treatment response. Treatment outcomes of iSGS patients in an ongoing cohort study (Gelbard et al., 2018) demonstrate that the most durable treatment responses are seen with surgical resection of the affected airway scar and replacement with healthy proximal airway mucosa (an operation termed cricotracheal resection, CTR (Grillo, 1982) (Fig. 5A). 38 iSGS patients undergoing CTR were propensity score matched to 38 iSGS patients undergoing endoscopic dilation (patient characteristics described in supplemental table 4). CTR had a 5% recurrence rate (2/38), compared with 47% for endoscopic dilation (18/38).(Fig. 5B). Kaplan-Meier analysis and log rank testing confirm a significantly lower rate of disease recurrence in patients that underwent CTR (P < 0.0001). These data suggest restoring the epithelial barrier with healthy mucosa minimizes disease recurrence.

## DISSCUSSION

Idiopathic subglottic stenosis (iSGS) is a debilitating localized fibrosis of the proximal airway. Affected patients possess tightly conserved clinical demographics, histopathology, and physiologic impairment (Gelbard et al., 2019). Our human data suggest that defects in epithelial barrier function can allow displacement of the native microbial community deep into the airway mucosa and contribute to dysregulated immune activation. Our complementary animal data support the necessity of bacteria and adaptive immune response to the observed tissue remodeling after proximal airway epithelial injury. Clinical treatment outcome data from an international iSGS patient cohort suggests that restoring the epithelial barrier with healthy mucosa minimizes disease recurrence.

The pseudostratified epithelium lining the human airway comprises several distinct populations of cells with specialized effector functions. Airway epithelium and the overlying mucociliary layer maintain a physical barrier against environmental insults (pathogens, allergens, and toxins). Many primary respiratory diseases, including chronic obstructive pulmonary disease (COPD), asthma, and idiopathic pulmonary fibrosis (IPF), display substantial pathological alterations in the airway epithelium. Evidence suggests that impairment of the epithelial barrier allows bacteria to penetrate the overlying mucociliary layer, intercalate within the epithelium, and activate host immunity. When sustained inappropriately, this inflammation can culminate in fibrotic tissue remodeling and physiologic impairment.

In COPD secondary to cigarette exposure, respiratory mucosal inflammation and fibrotic remodeling contribute to small airway obstruction and clinical symptoms(McDonough et al., 2011). Yet even after smoking cessation, many patients with COPD have sustained inflammation and disease progression (Rutgers et al., 2000). Published data demonstrate that persistent epithelial dysfunction in COPD leads to bacterial invasion deep into the mucosa and fibrotic airway remodeling (Polosukhin et al., 2017). Animal models suggest that endogenous bacteria orchestrate a persistent and pathologic adaptive immune response that drives tissue remodeling(Richmond et al., 2021). Similarly, in idiopathic pulmonary fibrosis, animal models, *in vitro* human data and genetic evidence suggest that the airway epithelium plays a central role in disease susceptibility (Li et al., 2004; Seibold et al., 2011).

Beyond the central role airway epithelial cells play in human respiratory disease, numerous studies have shown a specific role for the basal cell subset in repair and regeneration of the respiratory system after epithelial injury (Smirnova et al., 2016; Voynow et al., 2005). Fate-mapped mouse strains have shown that basal cells contribute to regeneration of the pulmonary parenchyma in response to both chemical(Vaughan et al., 2015) and viral injury (Kumar et al., 2011). Murine models of chemical-induced epithelial injury have shown that severe mucosal destruction involving the basal cell layer is associated with uncontrolled proliferation of the underlying stroma, resulting in an accumulation of fibroblasts and immune cells that subsequently obliterate the airway lumen (O’Koren et al., 2013). The reduction of basal cell subsets we observed in our data may partially explain the tissue remodeling seen clinically in iSGS(Mark et al., 2008; Trevino-Villarreal et al., 2021).

Interestingly, in addition to the observed basal cell depletion in iSGS airway scar, geneset enrichment analysis (GSEA) of the residual epithelial cells showed a mechanistic target of rapamycin (mTORC1) pathway activation and enhanced aerobic metabolism. mTOR is a master sensor that integrates environmental factors to regulate cell growth. In general, activation of mTOR stimulates proliferation, mitochondrial biogenesis, and oxidative phosphorylation (Laplante and Sabatini, 2012). The GESA findings were consistent with the observed KI67 and CDK1 expression (marking cellular proliferation) in the epithelium of iSGS airway scar. Additionally, GSEA showed that the residual epithelium in iSGS airway scar acquired a mesenchymal phenotype with epithelial-mesenchymal transition (EMT) pathway enrichment. EMT allows the disassembly of cell-cell junctions, actin cytoskeleton reorganization, and induction of contractile proteins as non-motile epithelial surfaces convert into individual, motile mesenchymal phenotypic. These phenotypic alterations may result from localized pathogen-driven inflammation(Hofman and Vouret-Craviari, 2012) or can result from mucosal injury secondary to physiologically relevant bile acid exposure(Aldhahrani et al., 2018). Integrating the basal cell depletion with GSEA findings supports the hypothesis that mucosal barrier dysfunction participates in iSGS disease pathogenesis.

Although an often-overlooked anatomic subsite, the subglottis is uniquely enriched in antigen-presenting dendritic cells and T lymphocytes (Friedman et al., 2007; Jecker et al., 1999). Additionally, it functions as a transition zone from the ciliated lining of the trachea to the squamous epithelium of the larynx. As a consequence, the subglottis has increased exposure to pathogens as the cilia-driven upward movement of the airway mucus layer temporarily stalls (Lee et al., 2000). While the inciting event for barrier dysfunction in iSGS is unclear, both genetic predisposition and environmental insults can impair epithelial function. The genetic foundations of iSGS remain obscure (Drake et al., 2020). Neither an association of iSGS with HLA subtypes nor alternate genetic risk alleles have yet been explored.

Prior small case series examining environmental factors contributing to iSGS have implicated disruption of the proximal airway microbiome, one using pathogen-specific molecular approaches(Gelbard et al., 2017), and another employing 16S rRNA sequencing of mucosal swabs(Hillel et al., 2019). While these studies implicated the presence of microbial species in iSGS, our new data from a larger, and more diverse patient cohort provides an unbiased characterization of the mucosal tissue microbiome. Our current results suggest that rather than microbiome disruption, in iSGS a “healthy microbiome” is displaced across a dysfunctional epithelium. This displacement is associated with adaptive immune infiltration and activation in response to bacterial species. This is supported by the finding that both a matched host microbiome, as well as the microbiome from a healthy control can drive adaptive immune activation in the proximal airway scar.

However, the displaced microbiome may not be the sole target of the adaptive immune response observed in iSGS mucosal scar. A feed-forward inflammatory loop may become established when peptides from microbial proteins share suffi cient structural similarity with self-peptides and activate autoreactive T cells, termed “molecular mimicry”(Bachmaier et al., 1999). Inflammation resulting from bacterial infection can also activate local antigen-presenting cells and enhance the processing and presentation of self-antigens, referred to as “epitope spreading”. Alternatively, T cell populations can be activated to mount effector responses in an antigen-independent, inflammation-dependent manner (termed “bystander activation”). Sustained bystander activation of CD8 T cells has been shown to generate spatially restricted tissue damage in the human disease (Groh et al., 2003; Meresse et al., 2004).

Although we acknowledge the limited ability to assign causality in pathologic studies involving human tissue, single-cell transcriptional profiling provides an unbiased assessment of the cell types within the human subglottis and illuminates the epithelial and immune alterations accompanying disease. Histologic localization of pathogens deep in the lamina propria and bacterial community profiling support the functional impact of the observed epithelial dysfunction rather than suggest microbiome disruption is a primary driver of disease. Animal models confirm the importance of both bacteria and host immunity in the pathogenesis of airway fibrosis after epithelial injury and robust clinical data suggests restoring the epithelial barrier with healthy mucosa minimizes disease recurrence.

**Figure 6:**
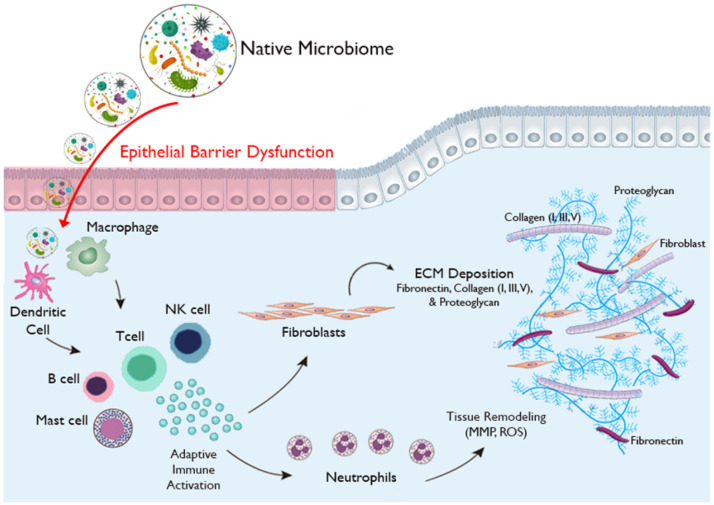
Proposed model of iSGS disease pathogenesis. Defects in epithelial barrier function allow translocation of the native microbial community deep into the airway mucosa and promote dysregulated immune activation leading to fibrotic remodeling and subsequent airway obstruction.

Despite the inherent limits involved in rare disease research, our findings dramatically shift our concept of iSGS disease pathogenesis (Fig. 6). Unbiased transcriptional data, functional studies and clinical data support the concept that epithelial barrier dysfunction allows translocation of native bacteria deep into the airway mucosa and drives dysregulated immune activation leading to fibrotic remodeling. The disease model establishes a new direction for future studies in iSGS. Treatments that noninvasively promote epithelial barrier integrity and blunt the local adaptive immune response may benefit patients and warrant rigorous future study.

## METHODS

Detailed experimental methods are included in the Supplemental Material.

## Data Availability

The accession number for the 16S sequencing data reported in this paper Bioproject number PRJNA784956, and the accession number for the single-cell RNA-sequencing data is GSE191128.
